# The food allergy COPE inventory: Adaptation and psychometric properties^☆^

**DOI:** 10.1016/j.waojou.2022.100626

**Published:** 2022-02-02

**Authors:** Gabriel Lins de Holanda Coelho, Paul H.P. Hanel, Aideen Byrne, Jonathan Hourihane, Audrey DunnGalvin

**Affiliations:** a Psychology, University College Cork, Ireland; b Psychology, University of Essex, United Kingdom; c Immunology, Crumlin Children's Hospital, Ireland; d Royal College of Surgeons, Ireland

**Keywords:** Coping strategies, Food allergy, Allergies, Psychometric

## Abstract

**Background:**

Food allergy (FA) has been shown to have an adverse impact on food allergy quality of life (FAQL). To more fully understand this impact, correlates and predictors of FAQL must be reliably measured. Coping is one such factor. In the present study (n = 200), we sought to adapt the widely used Coping Orientation to Problems Experienced (COPE) Inventory and its 15 distinct strategies to food allergy, named FA-COPE Inventory. More specifically, we propose a long (60-item) and short (30-item) version of the measure.

**Methods:**

To examine the robustness of the newly adapted FA-COPE Inventory, we tested whether the 15-factor structure of the adapted version would present good psychometrical properties, using gold standard psychometric techniques. We used Confirmatory Factor Analysis to assess model fit, McDonald's omega, and inter-item correlations to assess reliability, and Pearson's correlation to assess convergent validity with a generic coping measure and satisfaction with FA life.

**Results:**

Our results showed a good model fit (eg, CFI and TLI ≥ .94) for the 15-factor structure of the measure's long and short version. These factors also presented reliability levels aligned with the coping literature. Finally, the majority of the FA-COPE Inventory factors (eg, acceptance) were significantly associated with the generic coping measure and satisfaction with FA life.

**Conclusion:**

Both the long and short adapted FA-COPE Inventory showed a good fit to food allergy issues. These measures can help facilitate the identification of the most commonly used strategies to deal with FA. Their use can lead to a more in-depth understanding of the impact of the coping strategies and how they can help improve the quality of life of those impacted by the disease.

## Introduction

Food Allergy (FA) is responsible for approximately 90% of all allergic reactions worldwide,^1^ with an estimation of 240–550 million people affected by the disease.^2^ Having a FA can impact adversely on everyday life. Going out to a restaurant, being invited by friends for dinner, or simply getting the groceries becomes more stressful. The development of Patient Reported Outcome Measures (PROMS) such as Health Related Quality of Life (HRQL) disease-specific questionnaires ensure that the impact of food allergy (and any treatments provided) are evaluated from the perspective of the patient or parent. Since the use of HRQL measures in multiple settings has increased, including in clinical trials of new treatments, it is crucial to identify, describe, and define factors that may impact HRQL outcomes, so that they may be accounted for.

One potential factor is the type of coping strategy used by an individual. For instance, adaptive coping strategies may help mitigate the stress associated with FA and promote constructive management.^3^^,^^4^ In contrast, maladaptive coping strategies are more likely to lead to poor management and more adverse health and well-being outcomes.^5^ The Coping Orientation to Problems Experienced (COPE) Inventory is the most widely used and validated self-report measure to assess these different coping styles.^5^^,^^6^ The measure has been applied in diverse samples, including allergies,^7^ congenital heart disease,^8^ and other chronic diseases.^9^ Therefore, it is vital to accurately measure coping styles, specifically in food allergy, to target support where needed. Disease-specific measures are more sensitive than generic measures because the challenges associated with FA are distinct from other chronic illnesses.^10^

In the present research, we tested whether the 15 distinct coping strategies from the COPE inventory^5^ can be adapted to measure coping strategies to deal with food allergies. The assessment of coping strategies within the FA context helps with a more in-depth understanding of their efficacy and, therefore, ultimately increases the quality of life for people with the disease. More specifically, coping questionnaires typically assess how various strategies are used in the context of general stressful events. By adapting the instructions and items of an existing measure of coping strategies to food allergy, we hope to develop a questionnaire that is sensitive to patients with food allergy, resulting in a more robust interpretation of how different strategies are used. Food allergy researchers have emphasized the need to develop disease-specific measures. Indeed, several FA-specific questionnaires were developed or adapted over the years, such as the Food Allergy Quality of Life (FAQL)^11^ and the Food Allergy Anxiety Scale (FAAS).^12^ The development of the FA-COPE Inventory is part of the Food Allergy Coping and Emotions (FACES) project. FACES aims to assess the underlying social and psychological mechanisms of living with food allergy, especially identifying the different coping strategies used across age groups.

## Method

### Participants and procedure

Participants were 200 individuals (*M*_*age*_ = 35.81; *SD*_*age*_ = 12.77; Age range, 18–72; 138 women, 62 men) from the United Kingdom (n = 194) and Ireland (n = 4; missing = 2), recruited via Prolific (https://prolific.co/), a crowdsourcing platform for online studies.^13^ When setting up the study, we pre-screened participants with an approval rate of 98% and experience with previous studies on the platform, indicating reliable and high-quality data. The majority reported an allergy to peanuts (*n* = 62) and tree nuts (*n* = 50), were diagnosed in 2005 or before (*n* = 110), and mainly by a general practitioner∖family doctor (*n* = 73). Please, see the supplemental table for detailed information about the reported allergies and diagnosis.

### Material

The COPE Inventory^5^ comprises 60 items, equally distributed across 15 coping strategies (eg, active coping, seeking social support, focus on and venting of emotions). Shorter versions of the COPE were developed over the years, such as the Brief COPE,^6^ consisting of the 2 best items per factor from the original measure. Both the full COPE Inventory and its short versions have been validated in many countries, such as France,^14^ Estonia,^15^ Brazil,^16^ Croatia,^17^ China,^9^ and Greece.^18^ The coping strategies proposed by Carver^5^^,^^6^ were meaningfully associated with well-being variables across various contexts. For example, active and affective coping strategies were associated with lower levels of mental distress among bereaving parents.^19^ Moreover, among people working remotely during the spring 2020 COVID-19-induced lockdown, active coping was positively, and self-blame was negatively associated with well-being.^20^ These findings are supported by meta-analytic evidence.^21^ Active coping, planning, and acceptance strategies are usually among the most frequently used coping strategies.^6^^,^^22^

In the original COPE Inventory, the instructions refer to how respondents generally cope with stressful events. Thus, we first modified the questionnaire's instructions, informing participants that the items (ie, questions) refer to how they cope with their food allergy. Next, each of the items was amended to ensure that they were specific to food allergy. For example, the Item “*I concentrate my efforts on doing something about it*” (Active Coping) became “*I concentrate my efforts on doing something about my food allergy*”. The final version of the our Food Allergy COPE Inventory (FA-COPE Inventory) consisted of the same amount of items as the original version. Participants answer the frequency of applying each coping style, using a seven-point scale (1 = *Never*; 7 = *Every time*). The full measure is available as an appendix.

Next, we tested whether the factor structure of the adapted version of the 60-item COPE Inventory was replicated, using robust statistical analysis techniques, such as Confirmatory Factor Analysis (CFA) and convergent validity. First, we assessed whether the 15-dimension factorial structure of the FA-COPE Inventory would present a good model fit, like its original version. Then, we provided an alternative (and shorter) version, the Short FA-COPE Inventory, using the 2 items with the highest factorial loadings across all coping strategies. Shorter versions can help to provide faster and more accessible data collection, thereby reducing participants' boredom and fatigue.^23^ We assessed reliability for both full and short versions of the FA COPE Inventory, through McDonald's omega (ω; for the long version) and inter-item correlations (for the shorter version). Moreover, we explored which food allergy-specific coping strategies were most frequently used across both versions. Finally, we tested for convergent validity by analyzing the relations between the FA-COPE with a generic coping measure (see below), and one question asking how satisfied participants are with their current life with food allergy, from zero (*not at all*) to 10 (*completely*).

As stated above, we used a generic coping measure to assess convergent validity, the Brief Resilient Coping Scale.^24^ This measure comprises four items (eg*, I look for creative ways to alter difficult situations*). Participants answered to what extent the items describe them, using a five-poínt scale (1 = *Does not describe me at all*; 5 = *Describes me very well*).

### Data analyses

All analyses were performed using the statistical program *R*. For the Confirmatory Factor Analysis (CFA), we used the variance-adjusted weighted least squares (WLSMV) estimator and polychoric matrix. For model fit, the following indices were considered:^25^^,^^26^ (1) Comparative Fit Index (CFI) and (2) Tucker-Lewis Index (TLI), with suggested results over .90; and (3) Root mean square error approximation (RMSEA), with suggested results lower than .08 for a good fit. Moreover, to assess reliability, we used McDonald's omega (ω) for the full version of the FA-COPE Inventory, with results over .70 being adequate.^27^ Due to the low number of items per factor in the shorter version of the FA-COPE Inventory, we used inter-item correlations to assess whether the items are related to each other. Finally, Pearson's correlations were performed to assess the convergent validity of the COPE-FA.

## Results

### Confirmatory factor analysis and reliability

To assess the structure of the FA-COPE, we performed a confirmatory factor analysis (CFA). We followed the structure from the original COPE for our analyses, which is composed of 15 factors, with 4 items per factor. Results indicated a good overall model fit for this long version of the FA COPE Inventory: CFI = .94; TLI = .94; RMSEA = .049 (90%-CI = .045-.053). The factor weights for the FA-COPE ranged from .25 (Item 16, Mental Disengagement) and .99 (Item 18, Religious Coping). Table 1 shows a summary of the factorial loadings. We then tested an alternative model for a Short FA-COPE based on the factorial loadings, with the 2 best items composing each factor. The 30-item model also presented good overall model fit: CFI = .98; TLI = .97; RMSEA = .046 (90%-CI = .035 −.056)], with items ranging from .50 (Item 37, Behavioral Disengagement) and 1 (Item 48, Religious Coping; Item 50, Humor). All loadings in both models were statistically different from zero (λs ≠ 0; *Zs* > 1.96, *ps* < .01).

**Table 1:** Factorial loadings, reliability & means: long FA-COPE, short FA-COPE.

	Long FA-COPE			Short FA-COPE		
	λ (Range)	McDonald's omega ω	*M* (*SD*)	λs	Inter-item correlations	*M* (*SD*)
Acceptance	.66–.85	.79	5.61 (1.26)	.71, .91	.56∗	5.66 (1.43)
Active coping	.46–.73	.59	3.46 (1.17)	.72, .73	.41∗	2.51 (1.34)
Behavioral disengagement	.46–.80	.57	2.05 (0.88)	.50, .70	.22∗	1.9 (1)
Denial	.44–.97	.66	1.68 (0.84)	.76, .92	.50 ∗	1.52 (0.86)
Focus on and venting of emotions	.67–.86	.79	2.1 (1.01)	.82, .84	.58∗	1.85 (1.04)
Humor	.86–.96	.94	3.12 (1.6)	.86, 1	.87∗	3.16 (1.72)
Mental disengagement	.25–.78	.53	2.6 (1.06)	.78, .80	.51∗	2.01 (1.21)
Planning	.53–.79	.77	3.58 (1.33)	.72, .76	.51∗	3.36 (1.5)
Positive reinterpretation and growth	.68–.88	.81	2.91 (1.46)	.71, .83	.53∗	2.97 (1.6)
Religious coping	.92–.99	.95	1.52 (1.13)	.93, 1	.87∗	1.53 (1.2)
Restraint	.49–75	.64	2.59 (1.15)	.64, .68	.36∗	2.41 (1.26)
Substance use	.87–.96	.85	1.28 (0.63)	.96, .97	.80∗	1.21 (0.56)
Suppression of competing activities	.54–.84	.63	2.62 (1.11)	.72, .91	.55∗	2.08 (1.14)
Use of emotional social support	.60–.85	.83	2.6 (1.2)	.77, .81	.56∗	2.34 (1.25)
Use of instrumental social support	.78–.83	.83	2.4 (1.18)	.80, .84	.58∗	2.25 (1.26)

*Note*: λ = *Factorial Loadings;* ∗p < .*01*

Furthermore, we assessed the reliability for both long and short versions of the FA-COPE factors (Table 1). We used McDonald's omega for the long version of the measure, with the reliability levels ranging from .53 (Mental disengagement) to .95 (Religious Coping). The reliability of the full 60-item measure is .94. Moreover, we used inter-item correlation for the short FA-COPE Inventory. That is, the two items that compose each factor were correlated. All inter-item correlations were significant (*p* < .01), and ranged between .22 (Behavioral Disengagement) and .87 (Humor and Religious Coping).

### Frequencies

Table 1 also shows the means for each coping strategy of the long and short versions of the FA-COPE Inventory. For the long version, the most frequently used strategies in the sample were acceptance (*M* = 5.61), planning (*M* = 3.58), and active coping (*M* = 3.46). For the short version, the most frequently used strategies were acceptance (*M* = 5.66), planning (*M* = 3.36), and humor (*M* = 3.16). The least used strategies were denial (*M* = 1.68), religious coping (*M* = 1.52), and substance use (*M* = 1.28) for the long version. These were also the least used for the short version but in a different order: religious coping (*M* = 1.53), denial (*M* = 1.52), and substance use (*M* = 1.21). This similarity in frequencies between the long and short version further supports the validity of the short version.

### Convergent validity

**Fig. 1:**
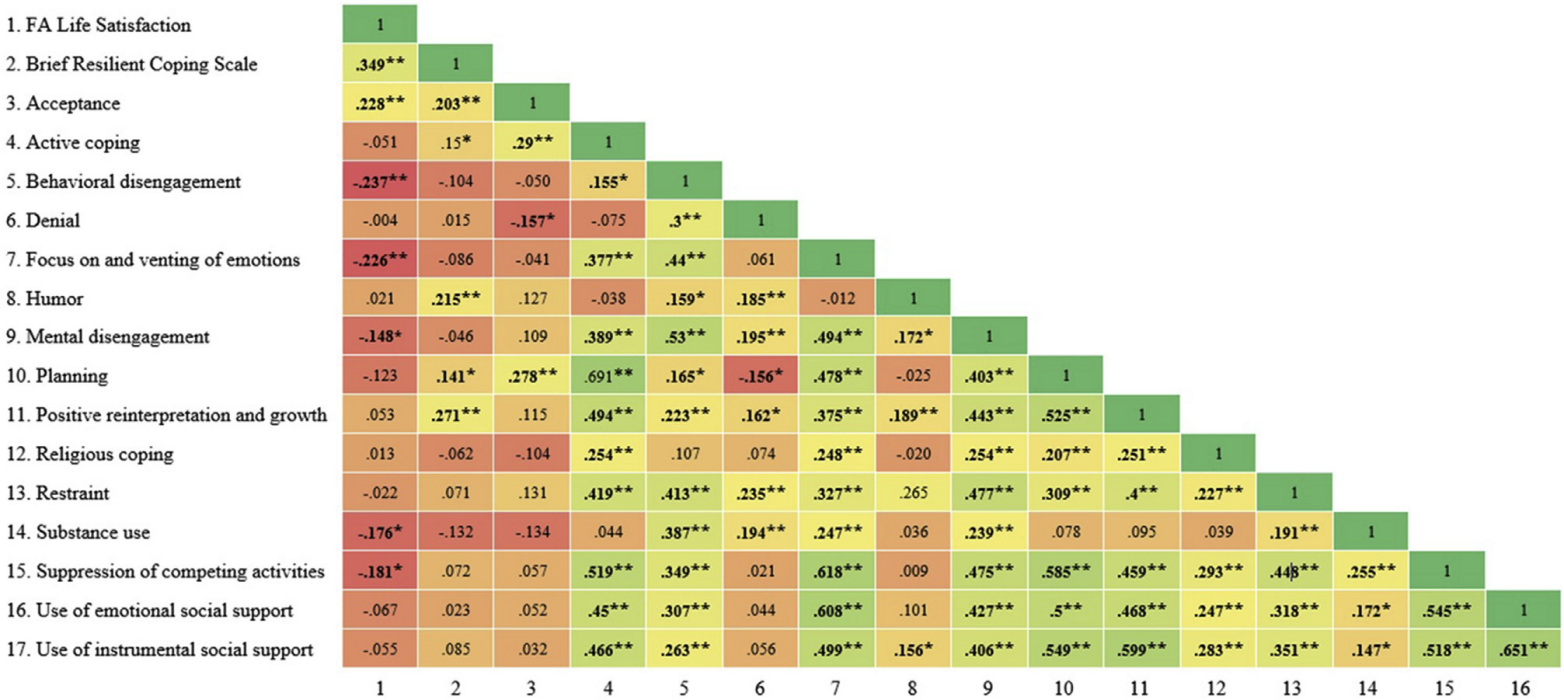
Correlation matrix: FA life satisfaction, brief resilience coping scale, and long FA-COPE factors

Fig. 1 shows the correlations between the FA-COPE Inventory with a generic coping measure and a single-item of satisfaction with FA life. The generic coping measure was significantly correlated with many FA-COPE factors: acceptance (*r* = .20, *p* < .01), active coping (*r* = .15, *p* < .05), humor *(r* = .21, *p* < .01), planning (*r* = .14, *p* < .05), and positive reinterpretation and growth (*r* = .27, *p* < .01). Moreover, satisfaction with FA life was significantly and positively associated with acceptance (*r* = .23, *p* < .01), and negatively with behavioral disengagement (*r* = −.24, *p* < .01), focus on and venting of emotions (*r* = −.23, *p* < .01), mental disengagement (*r* = −.15, *p* < .05), substance use (*r* = −.18, *p* < .05), and suppresion of competing activities (*r* = −.18, *p* < .05). Fig. 1 also shows the correlations between each of the FA-COPE factors

## Discussion

The daily life of those with FA can be affected in several ways, such as through feelings of tension and uncertainty.^28^ Clinical research has highlighted many coping strategies that can be used to deal with stressful situations,^5^ such as living with FA. In the present research, we adapted the most used questionnaire to assess individual coping strategies, the COPE Inventory. Our study sought to adapt the widely used COPE Inventory for use in food allergy, named FA-COPE Inventory. Moreover, we also proposed a short version for the measure. The measures can allow for a more in-depth understanding of how coping strategies are used to manage food allergy in real-world contexts.

### Psychometrical properties

The COPE Inventory 15-dimension structure is well known cross-culturally.^9^^,^^14^^,^^16^ To ensure that the structure of a FA-specific adaptation would hold, we performed a confirmatory factor analysis (CFA) with results showing a good model fit. Because of the growing need for shorter instruments in applied settings,^23^^,^^29^ we also proposed the short FA-COPE Inventory, by selecting the 2 items with the highest factorial loadings. The CFA results were consistent with the long version, indicating a good overall model. Our results highlight the robust structure of the COPE Inventory, even when adapted for a different setting such as food allergy.

Moreover, we assessed the reliability levels for each of the long and short FA-COPE coping strategies, using McDonald's omega and inter-item correlations. The inter-item correlations were chosen for the short FA-COPE, as its low number of items per factor could influence analyses such as McDonald's omega. For the long version, the omega reliability levels ranged from .53 to .95. Even though some were lower than recommended (.70; ^27^), these values are consistent with other measures of coping. For instance, in the original development study, Carver et al^5^ found reliability levels ranging from .45 to .92. These results are also consistent in other studies. Fontaine et al.^30^ found values ranging from .39 to 91. These lower values were also observed cross-culturally. For instance, in Estonia, values ranged from .49 to .95, ^15^ whereas in Croatia, values ranged from .52 to .87.^17^ One possible explanation for these lower values is the small number of items used in each coping strategy. Skinner et al^31^ argue that 5 to 6 items per factor would help to produce acceptable reliability values. Importantly, these lower values should not be used to undermine the measure, which is known as a gold standard for assessing coping strategies and used across many countries and contexts. For the short version of the FA-COPE Inventory, all factors presented significant correlations, ranging from .22 to .87.

### Frequencies and convergent validity

In line with previous research,^6^^,^^22^ active coping, planning, and acceptance were the strategies most commonly used in our study. Both active coping and planning are problem-focused.^5^ Individuals who use these strategies tend to think ahead to manage a specific problem.^32^ This makes sense in the context of food allergy, where planning ahead (for eating out, shopping, traveling, and socializing) is necessary for many to stay safe. On the other hand, acceptance is an emotion-focus coping strategy.^5^ This is an essential asset for anyone with a chronic disease, including food allergy. When facing FA, individuals might experience many different emotion-inducing situations, positively and negatively, which could lead to psychological distresses, such as anxiety^12^ and depression.^33^ Therefore, using an emotion-focused coping strategy such as acceptance can teach participants how to live with the food allergy and its unpredictability.

Furthermore, we assessed the convergent validity of the long version of the FA-COPE Inventory, associating its factors with a generic coping measure (Brief Resilience Coping Scale), and an item assessing their life satisfaction while with FA. The generic coping measure was significantly associated with active coping and planning (problem-focused strategies), acceptance and positive reinterpretation (emotion-focused strategies), and humor. These results indicate that the adapted FA-Cope Inventory presents satisfactory convergent validity, measuring what it is supposed to measure.

Finally, we found significant bi-directional associations between the long FA-COPE Inventory factors and the single-item satisfaction with FA life. For instance, respondents with a higher level of acceptance (emotion-focused) also had higher satisfaction with FA life. Further support is given by the negative associations found between the single-item satisfaction with FA life and more maladaptive strategies,^32^ such as behavioral disengagement, focus on and venting of emotions, mental disengagement, substance use, and suppression of competing activities. Most of these strategies are known as “less useful”, and are characterized by giving up on the problem or moving away from it.^32^

### Future studies, limitations and final considerations

Future studies can further assess the psychometrical properties of the FA COPE Inventory. For instance, whether the structure also presents a good model fit and reliability across different countries. Moreover, future studies could assess whether the measure is psychometrically suitable for other age groups (ie, teenagers, children), through assessing its structure, and by testing for measurement invariance. Such analyses are necessary to guarantee whether the FA-COPE can be reliably used for these different groups and whether they similarly understand and answer the measure. Also, whereas our results were in line with the literature,^6^^,^^22^ with active coping, planning, and acceptance as the most used coping strategies by adults affected by food allergy, new studies would allow us to assess whether children and teenagers use different coping strategies and check their efficacy over time. Additionally, future studies could assess whether comorbidities or chronic conditions moderate the link between coping strategies and quality of life (eg, if certain coping strategies are more effective for some patient groups than for others) which would allow to make more tailored recommendations. However, as our measure is focused on FA, it is unlikely that these health conditions would influence the psychometric properties of the FA-COPE Inventory.

Finally, future studies can assess the impact of applying these coping strategies daily to deal with FA, and how these influence quality of life variables. Many socio-psychological variables (eg, the threat of exposure, need for vigilance) can seriously impact food allergy quality of life.^34^ Therefore, those affected by FA can use different coping strategies to deal with these diverse variables to achieve a higher quality of life and minimize the impact of the disease. For instance, is it possible that using active coping leads longitudinally to higher levels of well-being? Further, can strategies such as acceptance and humor lead individuals to perceive their lives as “more normal”? Identifying coping strategies that have a more substantial influence on the quality of life is crucial for developing interventions that help increase adaptive responses to food allergy, thereby reducing the use of maladaptive strategies. In FA research, several disease-focused quality of life questionnaires have been developed over the years,^34^ such as the Food Allergy Quality of Life Questionnaire, which is available for many age groups.^11^^,^^35^ Therefore, longitudinally assessing these relations between coping strategies and FA quality of life is essential for future research

To conclude, adapting the well-known COPE Inventory to food allergy is an essential step in FA research, which helps to identify the most commonly used strategies to deal with the disease. These patient-related outcome measures (PROMs) are increasingly used in practice, research, and clinical trials, with National Institute for Health and Care Excellence (NICE) and the US Food and Drug Administration (FDA) emphasizing their importance. Also, these disease-specific measures can help assess the disease's unique impact on the studied variable. Therefore, it is crucial to specify their assessment as a consequence of FA when assessing coping strategies, leading to more reliable results and novel insights.

## Abbreviations

Food allergy (FA), Food Allergy and Anxiety Scale (FAAS) Food Allergy Quality of Life (FAQL), Patient Reported Outcome Measures (PROMS), Health Related Quality of Life (HRQL), COPE (Coping Orientation to Problems Experienced), Food Allergy Coping and Emotions (FACES).

## Ethical approval

All procedures performed in this study involving human participants were in accordance with the 1975 Helsinki Declaration. This study was approved by the ethics committee of the School of Applied Psychology, University College Cork.

## Informed consent

Informed consent was obtained from all participants.

## Consent for publication

All authors provided input into the manuscript, reviewed the final draft and provided consent for publication.

## Authors contributions

Drs Gabriel Coelho and Paul Hanel wrote the first draft of the manuscript, which was then reviewed, amended, and approved by all co-authors.

## Funding

This project is funded through the 10.13039/100014364National Children's Research Centre, Paediatric Research Project Grants 2018 (C/18/12).

## Data availability

Derived data supporting the findings of this study are available from the corresponding author upon request.

## Declaration of competing interest

The authors have no conflict of interest to declare.

## References

[1] Cohut M. (2019). https://www.medicalnewstoday.com/articles/324094.

[2] World Allergy Organization (2013). https://www.worldallergy.org/UserFiles/file/WhiteBook2-2013-v8.pdf.

[3] Koeske G.F., Kirk S.A., Koeske R.D. (1993). Coping with job stress: which strategies work best?. J Occup Organ Psychol.

[4] Polloni L., DunnGalvin A., Ferruzza E. (2017). Coping strategies, alexithymia and anxiety in young patients with food allergy. Allergy.

[5] Carver C.S., Scheier M.F., Weintraub J.K. (1989). Assessing coping strategies: a theoretically based approach. J Pers Soc Psychol.

[6] Carver C.S. (1997). You want to measure coping but your protocol’ too long: consider the brief cope. Int J Behav Med.

[7] Knibb R.C., Horton S.L. (2008). Can illness perceptions and coping predict psychological distress amongst allergy sufferers?. Br J Health Psychol.

[8] Rychik J., Donaghue D.D., Levy S. (2013). Maternal psychological stress after prenatal diagnosis of congenital heart disease. J Pediatr.

[9] Tang W.P.Y., Chan C.W.H., Choi K.C. (2021). Factor structure of the brief coping orientation to problems experienced inventory in Chinese (Brief-COPE-C) in caregivers of children with chronic illnesses. J Pediatr Nurs.

[10] Greenhawt M. (2016). Food allergy quality of life and living with food allergy. Curr Opin Allergy Clin Immunol.

[11] Flokstra-de Blok B.M.J., DunnGalvin A., Vlieg-Boerstra B.J. (2008). Development and validation of the self-administered food allergy quality of life questionnaire for adolescents. J Allergy Clin Immunol.

[12] Coelho GL. de H., Byrne A., Hourihane J., DunnGalvin A. (2021). Development of the food allergy anxiety scale in an adult population: psychometric parameters and convergent validity. J Allergy Clin Immunol Pract.

[13] Palan S., Schitter C. (2018). Prolific.ac—a subject pool for online experiments. J Behav Exp Finance.

[14] Baumstarck K., Alessandrini M., Hamidou Z., Auquier P., Leroy T., Boyer L. (2017). Assessment of coping: a new French four-factor structure of the brief COPE inventory. Health Qual Life Outcome.

[15] Kallasmaa T., Pulver A. (2000). The structure and properties of the Estonian COPE inventory. Pers Indiv Differ.

[16] Brasileiro S.V., Orsini M.R.C.A., Cavalcante J.A. (2016). Controversies regarding the psychometric properties of the brief COPE: the case of the Brazilian-Portuguese version “COPE breve. PLoS One.

[17] Hudek-Knežević J., Kardum I., Vukmirović Ž. (1999). The structure of coping styles: a comparative study of Croatian sample. Eur J Pers.

[18] Kapsou M., Panayiotou G., Kokkinos C.M., Demetriou A.G. (2010). Dimensionality of coping: an empirical contribution to the construct validation of the brief-COPE with a Greek-speaking sample. J Health Psychol.

[19] Murphy S.A., Johnson C., Lohan J. (2003). The effectiveness of coping resources and strategies used by bereaved parents 1 and 5 Years after the violent deaths of their children. Omega J Death Dying.

[20] Russo D., Hanel P.H.P., Altnickel S., van Berkel N. (2021). Predictors of well-being and productivity among software professionals during the COVID-19 pandemic – a longitudinal study. Empir Software Eng.

[21] Webb T.L., Miles E., Sheeran P. (2012). Dealing with feeling: a meta-analysis of the effectiveness of strategies derived from the process model of emotion regulation. Psychol Bull.

[22] Acquadro Maran D., Varetto A., Zedda M., Ieraci V. (2015). Occupational stress, anxiety and coping strategies in police officers. Occup Med.

[23] Coelho G.L.H., Hanel P.H.P., Wolf L.J. (2020). The very efficient assessment of need for cognition: developing a six-item version. Assessment.

[24] Sinclair V.G., Wallston K.A. (2004). The development and psychometric evaluation of the brief resilient coping scale. Assessment.

[25] Hair JFJr, Black W.C., Babin B.J., Anderson R.E. (2015). Multivariate Data Analysis.

[26] Tabachnick B.G., Fidell L.S. (2013). Using Multivariate Statistics.

[27] Kline P. (2013).

[28] Spielberger C.D. (2010). The Corsini Encyclopedia of Psychology.

[29] Monteiro R.P., Coelho GL. de H., Hanel P.H.P., de Medeiros E.D., da Silva P.D.G. (April 27, 2021). The efficient assessment of self-esteem: proposing the brief rosenberg self-esteem scale. Appl Res Qual Life.

[30] Fontaine K.R., Manstead A.S., Wagner H. (1993). Optimism, perceived control over stress, and coping. Eur J Pers.

[31] Skinner E.A., Edge K., Altman J., Sherwood H. (2003). Searching for the structure of coping: a review and critique of category systems for classifying ways of coping. Psychol Bull.

[32] Litman J.A. (2006). The COPE inventory: dimensionality and relationships with approach- and avoidance-motives and positive and negative traits. Pers Indiv Differ.

[33] Goodwin R.D., Rodgin S., Goldman R. (2017). Food allergy and anxiety and depression among ethnic minority children and their caregivers. J Pediatr.

[34] Antolín-Amérigo D., Manso L., Caminati M. (2016). Quality of life in patients with food allergy. Clin Mol Allergy.

[35] de Blok B.M.J.F., Meulen G.N.V.D., DunnGalvin A. (2009). Development and validation of the food allergy quality of life questionnaire – adult form. Allergy.

