# Supplementary material for: The food allergy COPE inventory: Adaptation and psychometric properties
Source: World Allergy Organ J. 2022 Feb 2;15(2):100626. doi: 10.1016/j.waojou.2022.100626 (PMC8819115; doi:10.1016/j.waojou.2022.100626)
Supplement: Multimedia component 1 [file mmc1.docx]

**COPE FA**

The following questions ask how you have sought to cope with your food allergy. Read the statements and indicate the frequency that you have been using each coping style.

Never

Rarely

Occasionally

Sometimes

Frequently

Usually

Every time

1. I try to grow as a person as a result of my food allergy. **

2. I turn to work or other substitute activities to take my mind of my food allergy. **

3. I get upset when I think about my food allergy and let my emotions out. **

4. I try to get advice from someone about what to do with my food allergy.

5. I concentrate my efforts on doing something about my food allergy. **

6. I say to myself "my food allergy isn't real." **

7. I put my trust in God.

8. I laugh about my food allergy.

9. I admit to myself that I can't deal with having a food allergy. **

10. I restrain myself from doing anything too quickly about my food allergy. **

11. I discuss my feelings with someone.

12. I use alcohol or drugs to make myself feel better.

13. I get used to the idea that I have a food allergy.

14. I talk to someone to find out more about my food allergy. **

15. I keep myself from getting distracted by other thoughts or activities.

16. I daydream about things other than my food allergy.

17. I get upset, and am really aware of it.

18. I seek God's help. **

19. I make a plan of action.

20. I make jokes about my food allergy. **

21. I accept that I am allergic to food and that it can't be changed.

22. I hold off doing anything about my food allergy until the situation permits.

23. I try to get emotional support from friends or relatives. **

24. I just give up trying to reach my goal.

25. I take additional action to try to get rid of my food allergy. **

26. I try to lose myself for a while by drinking alcohol or taking drugs.

27. I refuse to believe that I have food allergy.

28. I let my feelings out.

29. I try to see my food allergy in a different light, to make it seem more positive.

30. I talk to someone who could do something concrete about my food allergy. **

31. I sleep more than usual.

32. I try to come up with a strategy about what to do about my food allergy. **

33. I focus on dealing with my food allergy, and if necessary, let other things slide a little. **

34. I get sympathy and understanding from someone.

35. I drink alcohol or take drugs, in order to think about my food allergy less. **

36. I kid around about my food allergy.

37. I give up the attempt to get what I want. **

38. I look for something good in my food allergy.

39. I think about how I might best handle my food allergy.

40. I pretend that my food allergy hasn't really happened. **

41. I make sure not to make matters worse by acting too soon about my food allergy.

42. I try hard to prevent other things from interfering with my efforts at dealing with my food allergy.

43. I go to movies or watch TV, to think about my food allergy less. **

44. I accept the reality of the fact that I am now allergic to food. **

45. I ask people who have had similar experiences with food allergy what they did.

46. I feel a lot of emotional distress because of my food allergy, and I find myself expressing those feelings a lot. **

47. I take direct action to get around my food allergy.

48. I try to find comfort in my religion. **

49. I force myself to wait for the right time to do something about my food allergy. **

50. I make fun of my food allergy. **

51. I reduce the amount of effort I'm putting into dealing with my food allergy.

52. I talk to someone about how I feel about my food allergy. **

53. I use alcohol or drugs to help me get through my food allergy. **

54. I learn to live with my food allergy. **

55. I put aside other activities in order to concentrate on my food allergy. **

56. I think hard about what steps to take about my food allergy. **

57. I act as though my food allergy hasn't even happened.

58. I do what has to be done about my food allergy, one step at a time.

59. I learn something from my food allergy. **

60. I pray more than usual.

------------------------------------------------------------------------

Scales (sum items listed, with no reversals of coding):

Positive reinterpretation and growth: 1**, 29, 38, 59**

Mental disengagement: 2**, 16, 31, 43**

Focus on and venting of emotions: 3**, 17, 28, 46**

Use of instrumental social support: 4, 14**, 30**, 45

Active coping: 5**, 25**, 47, 58

Denial: 6**, 27, 40**, 57

Religious coping: 7, 18**, 48**, 60

Humor: 8, 20**, 36, 50**

Behavioral disengagement: 9**, 24, 37**, 51

Restraint: 10**, 22, 41, 49**

Use of emotional social support: 11, 23**, 34, 52**

Substance use: 12, 26, 35**, 53**

Acceptance: 13, 21, 44**, 54**

Suppression of competing activities: 15, 33**, 42, 55**

Planning: 19, 32**, 39, 56**

**** = SHORT VERSION ITEMS**
