# Supplementary material for: The food allergy COPE inventory: Adaptation and psychometric properties
Source: World Allergy Organ J. 2022 Feb 2;15(2):100626. doi: 10.1016/j.waojou.2022.100626 (PMC8819115; doi:10.1016/j.waojou.2022.100626)
Supplement: Multimedia component 2 [file mmc2.docx]

| **Supplemental Table 1**  *Allergy Characteristics of Sample* | | |
| --- | --- | --- |
| *Type of Allergy* | *n* | % |
| Cow's Milk | 37 | 18.5% |
| Eggs | 28 | 14% |
| Tree Nuts | 50 | 25% |
| Peanuts | 62 | 31% |
| Shellfish | 44 | 22% |
| Wheat | 15 | 7.5% |
| Soy | 16 | 8% |
| Fish | 27 | 13.5% |
| Other | 74 | 37% |
| *When diagnosed* |  |  |
| Between 2006 and 2019 | 90 | 45% |
| 2005 or Before | 110 | 55% |
| *Diagnosed by* |  |  |
| Allergist | 48 | 24% |
| Paediatric Specialist | 18 | 9% |
| General Practitioner \ Family Doctor | 73 | 36.5% |
| Allergy Nurse | 6 | 3% |
| Dietician | 4 | 2% |
| Alternative Practitioner | 4 | 2% |
| Not formally diagnosed | 44 | 22% |
| Other | 3 | 1.5% |
